# A Comparative Study of Clinical vs. Digital Exophthalmometry Measurement Methods

**DOI:** 10.1155/2020/1397410

**Published:** 2020-03-23

**Authors:** Tháıs de Sous Pereira, Cristina Hiromi Kuniyoshi, Cristiane de Almeida Leite, Eloisa M. M. S. Gebrim, Mário L. R. Monteiro, Allan C. Pieroni Gonçalves

**Affiliations:** ^1^Laboratory of Investigation in Ophthalmology (LIM 33), Division of Ophthalmology, University of São Paulo Medical School, São Paulo, Brazil; ^2^Department of Radiology, University of São Paulo Medical School, São Paulo, Brazil

## Abstract

**Background:**

A number of orbital diseases may be evaluated based on the degree of exophthalmos, but there is still no gold standard method for the measurement of this parameter. In this study we compare two exophthalmometry measurement methods (digital photography and clinical) with regard to reproducibility and the level of correlation and agreement with measurements obtained with Computerized Tomography (CT) measurements.

**Methods:**

Seventeen patients with bilateral proptosis and 15 patients with normal orbits diseases were enrolled. Patients underwent orbital CT, Hertel exophthalmometry (HE) and standardized frontal and side facial photographs by a single trained photographer. Exophthalmometry measurements with HE, the digital photographs and axial CT scans were obtained twice by the same examiner and once by another examiner. Pearson correlation coefficient (PCC) was used to assess correlations between methods. Validity between methods was assessed by mean differences, interintraclass correlation coefficients (ICC's), and Bland–Altman plots.

**Results:**

Mean values were significantly higher in the proptosis group (34 orbits) than in the normal group (30 orbits), regardless of the method. Within each group, mean digital exophthalmometry measurements (24.32 ± 5.17 mm and 18.62 ± 3.87 mm) were significantly greater than HE measurements (20.87 ± 2.53 mm and 17.52 ± 2.67 mm) with broader range of standard deviation. Inter-/intraclass correlation coefficients were 0.95/0.93 for clinical, 0.92/0.74 for digital, and 0.91/0.95 for CT measurements. Correlation coefficients between HE and CT scan measurements in both groups of subjects (*r* = 0.84 and *r* = 0.91, *p* < 0.05) were greater than those between digital and CT scan measurements (*r* = 0.61 and *r* = 0.75, *p* < 0.05). On the Bland–Altman plots, HE showed better agreement to CT measurements compared to the digital photograph method in both groups studied.

**Conclusions:**

Although photographic digital exophthalmometry showed strong correlation and agreement with CT scan measurements, it still performs worse than and is not as accurate as clinical Hertel exophthalmometry. This trail is registered with NCT01999790.

## 1. Background

Exophthalmometry is the assessment of the anteroposterior position of the globe in the orbit relative to the orbital rim. Though a number of orbital diseases may be evaluated based on the degree of exophthalmos, there is still no gold standard method for the measurement of this parameter. One of the most widely used methods is Hertel exophthalmometry (HE) [1–3]. However, despite the ease and convenience afforded by this method, the problems of reproducibility and standardization of measurements remain unsolved [1, 3, 4].

As shown by several authors, computed tomography (CT) correlates well with HE while providing greater accuracy, [5–8] but the high cost, exposure to radiation, and the need for repeated measurements severely restrict the use of this technology for routine exophthalmometry.

Digital photography is a simple, noninvasive method of measurement, with the additional advantage that measurements are documented and the images are easily shared online. Photographic techniques have been used to evaluate palpebral position, [9, 10] but to our knowledge, only one previous study has compared the methods of photography, clinical exophthalmometry, and CT in the measurement of exophthalmos. The authors found the correlation between the methods to be weak, possibly due to the fact that the study was multicentric and involved different photographers and evaluators, compromising reliability and reproducibility [8].

In the present investigation, we compared digital and clinical exophthalmometry to radiological exophthalmometry (CT) in a sample of patients from a single center, employing a single trained photographer. We also determined the level of agreement and reproducibility of the respective measurements.

## 2. Methods

This was an observational, cross-sectional, and descriptive study conducted at a hospital-based tertiary-level ophthalmology and otorhinolaryngology outpatient clinic in São Paulo, Brazil. The study protocol complied with the tenets of the Declaration of Helsinki, was approved by the Institutional Review Board of the University of São Paulo Medical School, and all participants gave their informed written consent.

Between May 2016 and August 2017, 17 patients with bilateral proptosis defined as clinical exophthalmometry readings greater than 20 mm due to orbital diseases were recruited. We also recruited 15 patients with normal orbits who had recently been submitted to CT scanning for nasal evaluation. The inclusion criteria were: (i) age above 21 years, (ii) absence of ocular abnormalities such as degenerative myopia, microphthalmos, and anophthalmic socket, (iii) absence of orbital abnormalities such as previous fractures and congenital defects, (iv) good patient collaboration, and (v) absence of previous orbital, strabismus, or eyelid surgery. Patients with poor-quality images (head tilted or eyes not in primary gaze) were excluded.

### 2.1. Clinical Exophthalmometry

Patients were submitted to clinical exophthalmometry twice by one examiner (senior faculty) and once by a second examiner (senior faculty). All measurements were taken in the primary position, with the patient standing up and with the eyes at the same level as the examiner's eyes. The exophthalmometer (Oculus Inc., Dutenhofen, Germany) was fitted with a double mirror, without prism. The base was recorded and maintained at all 3 measurements.

### 2.2. Radiological Exophthalmometry

**Figure 1:**
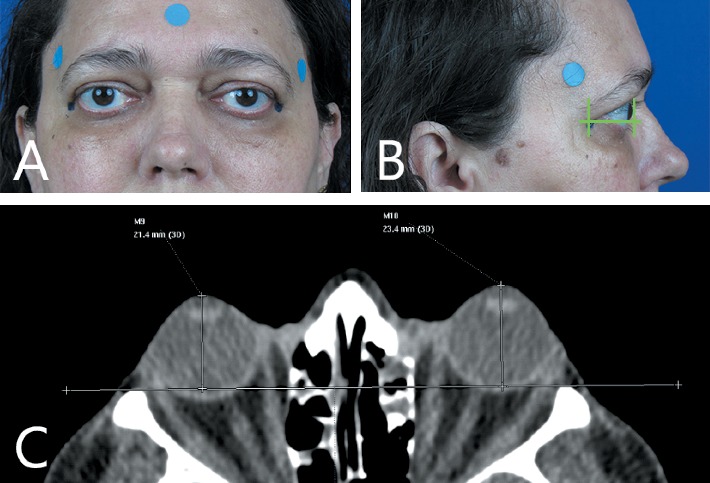
(a) Standardized frontal patient photography. (b) Side view photography with digital measurement method. (c) Radiological exophthalmometry method.

No later than 4 weeks after the ophthalmologic examination, patients were submitted to multidetector CT scanning of the orbit (Brilliance 16, Philips Medical Systems, the Netherlands) without intravenous contrast. Axial scanning was performed with the patient in dorsal decubitus and with the head parallel to the Frankfurt plane. Patients were instructed to keep their eyes open and static in the primary position of gaze. The acquisition parameters were 120 kv, 200 mAs; detector setting 16 × 0.75 mm; slice thickness 1.5 mm; and increase 0.7 mm. After acquisition, images were processed and analyzed with the dedicated workstation software (Extended Brilliance Workspace (EBW) Philips Medical Imaging, Best, the Netherlands) The images were examined by a head-and-neck radiologist and by a second reader, both of whom were blinded to the clinical condition of the patient. Having selected the full-orbit image with the greatest intraocular lens thickness, a line was drawn from the zygomatic rhymes to the anterior surface of the cornea in order to measure exophthalmos (Figure 1(c)) [8, 11]. The images of the right and left orbits were evaluated independently. The reliability of the measurements was assessed by repeating them on the same CT images 6 months later.

### 2.3. Digital Photography Exophthalmometry

Standardized frontal and side-view photographs (Canon Power-Shot SX530 HS) of each subject were taken by a single trained ophthalmologist. The patient was positioned in a chair with a blue background, with the head aligned in primary gaze and parallel to the photographer (Figure 1(a)). A surgical pen mark was made on the anterior border of the lateral orbital rim at the height of the lateral canthus. The photograph also included a 12 mm diameter circular sticker for digital calibration. The digital images were processed and analyzed by two readers with the assistance of custom software developed at Matlab (MathWorks, Natick, MA) by Garcia and colleagues [12]. One of the readers repeated the measurements on a different day. Based on the side-view photograph, exophthalmos was defined as the distance from the lateral orbital rim to the corneal vertex (Figure 1(b)).

### 2.4. Statistical Analysis

The statistical analysis was performed in the R Language [13]. The data generated with the three methods of exophthalmometry (clinical, radiological, and digital) in both groups (normal and proptosis) were compared with paired *t* tests. Intraclass and interclass correlation coefficients (ICC) were used to assess the consistency or reproducibility of the measurements. Radiological (CT) was the modality to which the other two methods were compared. Pearson correlations coefficient was used to evaluate the association between the different modalities, while the agreement between clinical and digital to radiological exophthalmometry was assessed with Bland–Altman plots. The level of statistical significance was set at 5% (*p* < 0.05).

## 3. Results

Thirty-four orbits with proptosis and 30 orbits without proptosis (normal group) were included in the study.  Table 1 shows the clinical, radiological, and digital exophthalmometry measurements of the normal and proptosis group, expressed as mean values ± standard deviation (SD). Mean values were significantly higher in the proptosis group than in the normal group, regardless of the method. Within each group, clinical exophthalmometry yielded significantly smaller mean values (control −0.58 mm; proptosis −0.82 mm) than radiological exophthalmometry. Moreover, mean digital exophthalmometry values were significantly greater (control +0.52 mm; proptosis +2.63 mm) than mean radiological exophthalmometry values (*p*=0.276 and *p* < 0.05, respectively).

**Figure 2:**
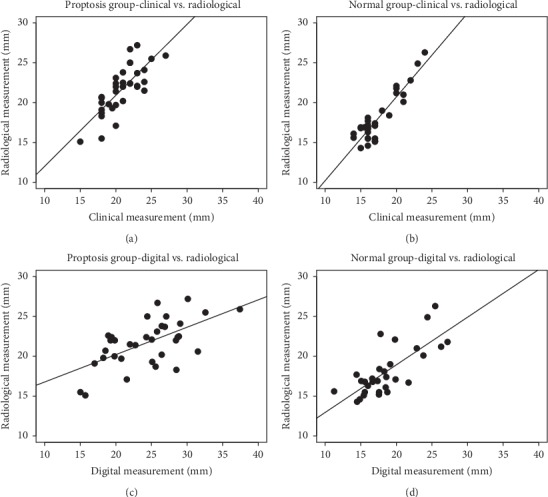
(a–d) Correlations between clinical and digital photographic measurements versus CT measurements.

The level of intra- and interclinician agreement of the clinical measurements was 0.93 and 0.95, respectively. The intra- and interclass correlation coefficients were, respectively, 0.95 and 0.91 for radiological measurements and 0.74 and 0.92 for digital measurements. A stronger correlation was observed between clinical and radiological measurements in both the proptosis group and the control group (Pearson correlation coefficient: *r* = 0.84 and *r* = 0.91, respectively; both *p* < 0.05). Somewhat weaker, although still significant, correlations were found between digital and radiological measurements (Pearson *r* = 0.61 and *r* = 0.75, respectively; both *p* < 0.05) (Figure 2).

**Figure 3:**
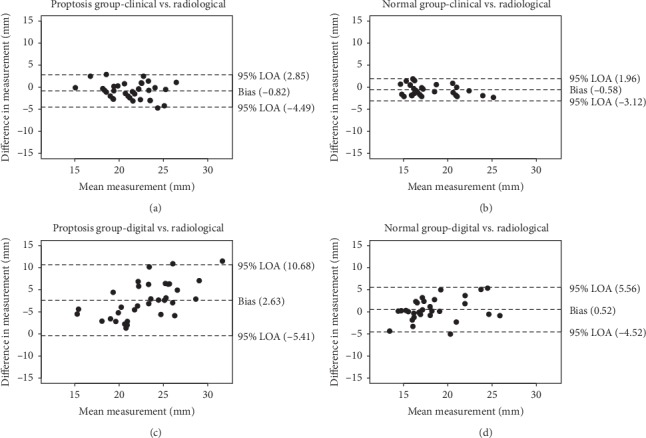
(a–d) Bland–Altman plots comparing clinical and digital photographic measurements versus CT measurements.

When comparing clinical and radiological measurements on the Bland–Altman plot [14], the 95% limits of agreement (LOA) were −3.12 and 1.96 mm for the control group and −4.49 and 2.85 mm for the proptosis group. While comparing digital and radiological measurements, LOA were −4.52 and 5.56 mm for the control group and −5.41 and 10.68 mm for the proptosis group. In other words, clinical measurements showed better agreement to CT especially in the normal orbits group (Figure 3).

## 4. Discussion

Exophthalmometry is an important tool in the evaluation and follow-up of orbital diseases. A variety of instruments have been used to measure proptosis [7, 15, 16], including radiological exophthalmometry, which was shown to correlate well with HE [6, 7, 16]. However, the use of CT solely for exophthalmometry should be avoided due to radiation exposure and high costs. HE is still the most widely used method, although readings, reproducibility, and accuracy are affected by the device type and examiner skill. In our study, the readings were made by a single senior faculty and repeated by another senior faculty in order to calculate interobserver variability [1, 4]. With all three measuring methods, mean values were significantly higher in the proptosis group than in the control group. In addition, accuracy remained unchanged as the degree of proptosis increased. Previous studies have yielded inconsistent results in this regard: some have found accuracy to be negatively associated with the degree of proptosis [2, 17], while others have not [8]. The high levels of reliability and reproducibility observed in this study (ICC = 0.93 for clinical measurements; ICC = 0.95 for radiological measurements) match the results of several other studies [3, 4, 16].

Due to the high intra- and interclass correlation of the CT measurements (an indication of good reproducibility and consistency), CT was chosen as the gold standard to which the other methods were compared. CT also correlated well with HE. Both observations are compatible with those of previous studies [6–8, 16].

Because patients are in the supine position during CT imaging and standing upright during HE reading, CT measurements are expected to be lower than Hertel readings. However, the opposite was observed in our study. This nevertheless coincides with Bingham's findings for the Oculus exophthalmometer, the same device used in this study [8]. The lower estimation of the HE method may overcome the position bias on measurement. On the other hand, the digital measurements from photographs made in the upright position were higher than the radiological measurements.

Digital exophthalmometry is a recent method to measure the axial globe position. Some authors using photography to evaluate palpebral position have reported good results in patients with Graves Orbitopathy [9, 10]. The upsides of using photography instead of CT or Hertel exophthalmometry are noninvasiveness and high availability. The downsides are the requirement of good standardization, including gaze, light, and camera parameters (position, zoom, and aperture). Furthermore, different that HE, measurements are not prompt available.

We found lower intraclinician correlation for digital than clinical and radiological measurements, indicating lower reproducibility, possibly due to inaccurate rhyme edge markings or photography inconsistency. Digital measurements also displayed higher mean value, broader range and standard deviations (especially in the proptosis group), and lower Pearson correlation indices to CT, when compared with clinical measurements.

On the Bland–Altman plot, LOA was larger in the proptosis group than in the control group in all methods comparisons. It was also larger for digital vs. CT measurements than for clinical vs. CT measurements in both groups. These findings suggest that measurements were less reliable (greater variation) in the proptosis group and when using digital photography.

Our study was carried out in a single center, employing a single trained photographer and specific custom software for the measurements. It also has some limitations as small sample size. Albeit the correlation indices observed in our study were better than the indices reported elsewhere [8], the level of agreement was below our expectations.

## 5. Conclusions

Digital photography exophthalmometry was associated with greater variance, lower correlation, and agreement to radiological measurements when compared to clinical exophthalmometry. Inspite of improving photography standardization and measurement consistency, digital exophthalmometry is still not accurate enough to supplant Hertel exophthalmometry.

## Figures and Tables

**Table 1:** Clinical, radiological, and digital exophthalmometry means and standard deviations (SD) for each group (normal and proptosis). Intra- and interclass correlation coefficients (ICC) of each exophthalmometry method.

Exophthalmometry method	Normal group 30 orbits	Proptosis group 34 orbits	ICC	
	Mean (range) ± SD (mm)	Mean (range) ± SD (mm)	Intra	Inter
Clinical (Hertel)	17.52 ± 2.67	20.87 ± 2.53	0.93	0.95
Radiological (CT)	18.10 ± 3.09	21.69 ± 2.91	0.95	0.91
Digital photography	18.62 ± 3.87	24.32 ± 5.17	0.74	0.92

## Data Availability

The datasets used and/or analyzed during the current study are available from the corresponding author on reasonable request. Requests for access to these data should be made to allanpieroni75@gmail.com.

## Abbreviations

HE: Hertel exophthalmometry
CT: Computed tomography
SD: Standard deviation
LOA: Limits of agreement
ICC: Intraclass and interclass correlation coefficients.
